# A preliminary report comparing the effect of Asafoetida with oral contraceptive on polycystic ovarian syndrome in a double-blind randomized trial

**DOI:** 10.22038/AJP.2023.23122

**Published:** 2024

**Authors:** Najmeh Dehparvar, Ahia Garshasbi, Amir Niasari-Naslaji, Fatemeh Alijaniha, Mohammad Gholami-Fesharaki, Farzaneh Ghaffari, Mohsen Naseri

**Affiliations:** 1 *Department of Traditional Persian Medicine, Faculty of Medicine, Shahed University, Tehran, Iran*; 2 *Department of Obstetrics and Gynecology, Medical School, Shahed University, Tehran, Iran*; 3 *Department of Theriogenology, Faculty of Veterinary Medicine, University of Tehran, Tehran, Iran*; 4 *Traditional Medicine Clinical Trial Research Center, Shahed University, Tehran, Iran*; 5 *Department of Biostatistics, Faculty of Medicine, Tarbiat Modares University, Tehran, Iran*; 6 *School of Traditional Medicine, Shahid Beheshti University of Medical Sciences, Tehran, Iran*; 7 *Department of Hikmat, Islamic and Traditional Medicine, The Academy of Medical Sciences, Tehran, Iran*

**Keywords:** Asafoetida, Infertility, Low-dose oral contraceptive PCOS, Persian medicine

## Abstract

**Objective:**

Polycystic ovarian syndrome (PCOS) is the most common cause of infertility and endocrine disorders in women of childbearing age. In Persian medicine, *Ferula assafoetida* L. (Asafoetida) was recommended for treating PCOS. The present study was conducted to compare the effect of Asafoetida with oral contraceptive tablets on PCOS patients.

**Materials and Methods:**

Patients with PCOS (n=30) were enrolled in a double-blind randomized clinical trial. On Day 5 of the menstrual cycle, patients received two periods of 21-day treatment, with 7 days rest between the two treatments. On a daily basis, half of the patients (n=15) received Asafoetida (1 g), and the rest received low dose oral contraceptive (LD; one tablet). Menstrual status, anthropometric characteristics, hematology and biochemistry parameters, ovarian ultrasound examination and hirsutism were evaluated prior to the initiation of the experiment and 14 days after the end of treatment. The occurrence of menstrual cycles and pregnancy was assessed eight months after the end of treatment.

**Results:**

The incidence of pregnancy was greater in patients who received Asafoetida compared to those who received LD (p=0.019). The time intervals between menstrual cycles became shorter in both groups (p<0.05). The occurrence of regular menstrual cycles remained longer in the Asafoetida compared to the LD group (p=0.001). Concentrations of triglycerides, cholesterol, HDL and LDL were significantly increased after treating with LD (p<0.05).

**Conclusion:**

In PCOS patients, the occurrence of regular menstrual cycles and the incidence of pregnancy were improved following treatment with Asafoetida. This medicament could be considered a safe treatment for patients with PCOS.

## Introduction

The most common cause of endocrine disorders and infertility among childbearing-aged women is polycystic ovarian syndrome (PCOS) (Gibbs et al., 2008; Liu et al., 2021). Its prevalence is 6 to 15% (Gibbs et al., 2008; Bozdag et al., 2016). PCOS could be associated with chronic anavoulatory status and polycystic ovaries (Gibbs et al., 2008). Increased oxidative stress (Artimani et al., 2018) following type II diabetes (Jahromi et al., 2021; livadas et al., 2022), obesity (Gibbs et al., 2008; Rosenfield, 2007) and chronic inflammation (Rudnicka et al., 2021; Rostamtabar et al., 2021; Keskin et al., 2014; Çakıroğlu et al., 2016) and decreased antioxidant concentrations (Palacio et al., 2006) could be considered risk factors for PCOS. Therefore, any type of medications that could reduce the incidence or intensity of these risk factors could be useful to assist patients with PCOS. 

Estrogen-progestin oral contraceptives are recommended for normalizing PCOS symptoms (Rosenfield, 2015); however, this medication could decrease insulin sensitivity (Diamanti-Kandarakis et al., 2003) and libido (Cooper et al., 2022), and increase triglyceride concentrations (Nader and Diamanti-Kandarakis, 2007), cardiovascular disease, breakthrough bleeding, nausea, headaches, abdominal cramping, breast tenderness, vaginal discharge, hypertension, and myocardial infarction and might have negative effect on the acquisition of bone mineral density (Cooper et al., 2022).

Persian medicine (PM) is an alternative approach to treat many medical conditions with special attention in the field of gynecology (Naseri et al., 2021; Goshtasebi et al., 2015; Mokaberinejad et al., 2014). Among various remedies recommended in PM for symptoms of PCOS, *Ferula assafoetida *L. (Asafoetda) is considered the most common one (Avicenna, 2005; Dehparvar et al., 2022). Asafoetida is an Iranian native plant that belongs to the Apiaceae family (Iranshahy and Iranshahi, 2011) which is the most used familiy to treat oligomenorrhea and amenorrhea in PM (Moini Jazani et al., 2018). Asafoetida is a herbal medicine with antioxidant (Amalraj and Gopi, 2017; Niazmand and Razavizadeh, 2021) and anti-inflammatory (Mahdavi et al., 2017; Shahrajabian et al., 2021) properties. It has positive effects against obesity (Amalraj and Gopi, 2017; Azizian et al., 2012) and it could reduce testosterone concentrations (Ayoubi et al., 2013). Asafoetida has hypolipidemic and antidiabetic activities and can normalize hyperglycemia and complications of diabetes (Latifi et al., 2019). Therefore, Asafoetida could be a good candidate for treating PCOS. The objective of this study was to investigate the effect of Asafoetida in comparison with low dose oral contraceptive (LD) on PCOS patients. 

## Materials and Methods

This study received an approval from Ethics Committee of Shahed University (IR.SHAHED.REC.1397.092; 2018-12-24) and was registered at the Iranian Clinical Trials Registry (IRCT20190728044360N1; www.irct.ir).

**Subjects**

Women at the age of 20-40 years, residents in Qom, Iran with symptoms of PCOS according to the Rotterdam criteria (ESHRE, 2004) were selected for this study by a gynecologist. Women with diabetes mellitus, thyroid disorders, diseases which could interact with PCOS such as hyperprolactinemia and women with sensitivity to oral contraceptives were excluded from the trial. Women were assigned into two groups (n=15 in each group) considering their age, BMI, education level, occupation and marital status (Table 1). Participants provided written consent prior to the initiation of the study.

**Study design**

Prior to the initiation of the experiment, demographic information, menstrual cycle status, anthropometric characteristics, hematology and biochemistry parameters, ovarian ultrasound examination and the presence of hirsutism were recorded. Hirsutism was evaluated by observing the presence of excess hair on the face and body with the male pattern.

In this double-blind randomized clinical trial, on Day 5 of the menstrual cycle (Day 0 of the experiment), patients received two periods of 21-day treatment, with 7 days rest period between two treatments. On a daily basis, patients in the herbal medicine group (n=15), received Asafoetida (1 g) and patients in conventional medicine group (n=15) received LD (one tablet (. 

**Medicine preparation**

Asafoetida (*Ferula assafoetida* L., herbarium number: PMP-888, Faculty of Pharmacy, Tehran University of Medical Sciences, Tehran, Iran) was encapsulated using oleo-gum resin (each capsule contained 500 mg Asafoetida) in Traditional Medicine Clinical Trial Research Center of Shahed University. Two capsules were administered everyday throughout the trial.

Standardization of Iranian Asafoetida, used in this study, was conducted to determine the total phenolic content based on gallic acid substance using Folin Ciocalteu reagent. The mean amount of total phenolic content in asafoetida was 4.15±0.26 mg gallic acid/g (Alijaniha et al., 2023).

LD tablet contained 0.03 mg Ethinyl Estradiol and 0.3 mg Norgestrel. In order to follow the double-blind design, patients in the LD group received two capsules similar to herbal medicine group. One capsule contained LD tablet in association with a bread powder and the other one contained just bread powder to have the same weight as Asafoetida capsule. 

Patient received 3 consultations of 20 min on Days 0, 28 and 56 of the experiment to seek their well-being and to assess possible adverse effects of medications. 

**Study outcomes**

The first assessment was conducted on Day 14 after the end of treatment to check any changes in the status of menstrual cycle, anthropometric characteristics, hematology and biochemistry parameters, ovarian ultrasound examination and hirsutism in comparison with initial assessments. The assessment of menstrual cycle status included the menstruation cycle length, the bleeding duration and the bleeding volume based on the number of sanitary napkin packages used during menstruation period. Anthropometric measurements included body weight (kg), body mass index (BMI), waist and hip circumferences (centimeter). Hematology and biochemistry analyses included total and free testosterone, hemoglobin, mean corpuscular volume (MCV), fasting blood sugar (FBS), triglyceride, cholesterol, high density lipoprotein (HDL), low density lipoprotein (LDL), aspartate aminotransferase (AST), alanine aminotransferase (ALT), and alkaline phosphatase (ALP). The size of ovary and the presence of ovarian cyst were evaluated using ultrasound examination. The presence of hirsutism was recorded.

The second assessment was conducted 8 months after the end of treatment by evaluating the regular occurrence of menstrual cycles and pregnancy status in married patients using Beta-hCG test. 

**Statistical analysis**

The statistical analysis was carried out using SPSS for Windows version 24 at a significance level of p<0.05. Qualitative and quantitative variables are reported as frequency (percent) and mean (±SD), respectively. Normal distribution of quantitative variables was checked using Kolmogorov Smirnov test. The difference between the two groups for quantitative variables was analyzed using T-test or Mann-Whitney U test. The difference between the two groups for qualitative variables was analyzed using Chi-square test. Paired sample T-test or Wilcoxon test was used to calculate matched samples.

## Results

**Figure 1:**
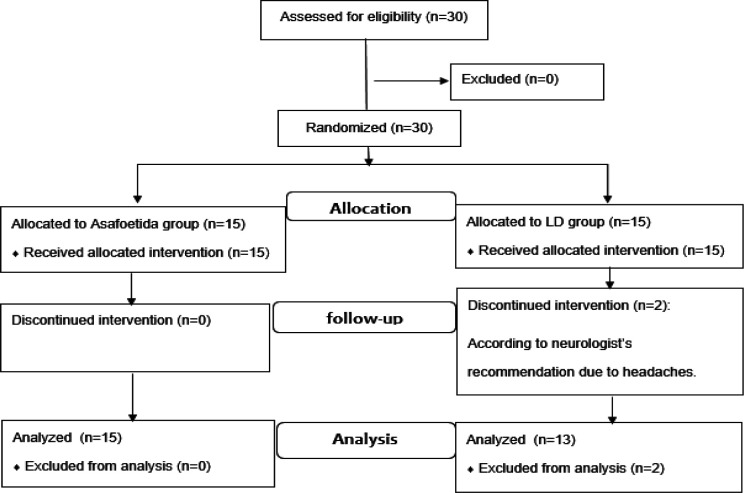
Consort flow diagram (enrollment analysis)

Figure 1 illustrates the Consort diagram of the present study. Two weeks after initiating the experiment, two patients receiving LD tablets displayed headache symptom. Therefore, according to the neurologist’s recommendation, they did not receive any further treatment and were excluded from the study. Ultimately, 28 patients completed the study and were analyzed (15 patients in the Asafoetida group and 13 patients in the LD group).

There were no significant differences between the two groups in demographical features (Table 1) or medical history (Table 2). The menstrual cycles interval in both groups reduced significantly after treatment (Table 3; p<0.05). No difference was found in menstrual cycle length within or between the two groups (Table 3; p>0.05). The bleeding volume was not significantly different within or between the two groups (Table 3; p>0.05). There was no significant alteration in anthropometric characteristics of the two groups when comparing before and after treatment (Table 4; p>0.05). The triglycerides, cholesterol, HDL and LDL concentrations in the LD group significantly increased after treatment (Table 5; p<0.05). Total testosterone tended to decrease in the LD group after treatment (Table 5; P=0.05). There was no significant difference in concentrations of other biomarkers particularly liver enzymes at the pre or post treatment between the two groups (Table 5; p>0.05). The ovarian ultrasound findings did not exhibit major differences between patients that received Asafoetida and those that received LD (Table 6; p=0.176). The presence of hirsutism was not significantly different within or between the two groups (Table 6; p>0.05).

Regular menstrual cycles maintained for a longer period in POCS patients treated with Asafoetida (6.5±2.95 months) compared to those who received LD (2.5±2.96 months; Table 3; p=0.001). The number of married patients who became pregnant within 8 months after the end of treatment, was greater in the Asafoetida group (7/14; 50%) compared to the LD group (0/10; 0%; Table 6; p=0.019).

**Table 1:** The demographical status of PCOS patients that received *Ferula assa-foetida *L (Asafoetida) and those who received low dose oral contraceptive (LD) treatments. Data are presented as No. (%).

**Variables**	**Status**	**Asafoetida** **(N=15)**	**LD** **(N=13)**	**p value**
Age (Y)	15-30	8 (53.3)	9 (69.2)	0.460
	30-45	7 (46.7)	4 (30.8)	
BMI (kg/m_2_)	< 25	3 (20.0)	4 (30.8)	0.352
	25 – 30	6 (40.0)	7 (53.8)	
	>30	6 (40.0)	2 (15.4)	
Education level	High school	4 (26.7)	6 (46.15)	0.498
	higher education	11 (73.3)	7 (53.85)	
Occupation	House-wife	12 (80.0)	10 (76.9)	0.999
	Other	3 (20.0)	3 (23.1)	
Marital status	Single	1 (6.7)	3 (23.1)	0.311
	Married	14 (93.3)	10 (76.9)	
The history of live birth	No	6 (42.9)	5 (50.0)	0.999
	Yes	8 (57.1)	5 (50.0)	
Contraception in married patients	No	6 (42.8)	5 (50.0)	0.629
	Yes	8 (58.2)	5 (50.0)	

**Table 2:** Medical history of PCOS patients that received *Ferula assa-foetida *L (Asafoetida) and those who received low dose oral contraceptive (LD) treatments.

**Variables **	** Status **	**Asafoetida ** **(N=15)**	**LD** ** (N=13)**	**p value**
Pregnancy history in married patients	No	5 (35.7)	4 (40.0)	0.999
	Yes	9 (64.3)	6 (60.0)	
Abortion history in married patients	No	11 (78.6)	9 (90.0)	0.615
	Yes	3 (21.4)	1 (10.0)	
Infertility history in married patients	No	12 (85.7)	6 (60.0)	0.192
	Yes	2 (14.3)	4 (40.0)	
Anemia history	No	8 (53.3)	7 (53.8)	0.999
	Yes	7 (46.7)	6 (46.2)	
Diabetes history	No	15 (100.0)	12 (92.3)	0.464
	Yes	0 (0.0)	1 (7.7)	
Hypertension history	No	14 (93.3)	12 (92.3)	0.999
	Yes	1 (6.7)	1 (7.7)	
Hyperlipidemia history	No	14 (93.3)	13 (100.0)	0.999
	Yes	1 (6.7)	0 (0.0)	
Hypothyroidism history	No	6 (40.0)	6 (46.2)	0.999
	Yes	9 (60.0)	7 (53.8)	
Inter menstrual bleeding	No	11 (73.3)	9 (69.2)	0.999
	Yes	4 (26.7)	4 (30.8)	

**Table 3:** The characteristics of menstrual cycles in PCOS patients that received *Ferula assa-foetida *L (Asafoetida) and those who received low dose oral contraceptive (LD) treatments. Data are presented as Mean±SD.

**Variables**	**Status**	**Group**	**Pre-treatment**	**Post-treatment**	**p value**	
					**With in**	**Between**
MCI (day)		asafoetida	138.0±100.01	32.1±5.80	0.001	0.210
		LD	90.8±47.52	29.1±1.07	0.002	
MCL (day)		asafoetida	7.1±1.71	6.7±1.86	0.391	0.693
		LD	7.0±1.22	6.9±1.38	0.798	
RMC (Month)		asafoetida		6.5±2.95		0.001
		LD		2.5±2.96		
Bleeding volume	Few	asafoetida	3 (20)	7 (46.6)	0.172	0.101
	Normal		11 (73.3)	7 (46.6)		
	Excessive		1 (6.7)	1 (6.7)		
	Few	LD	1 (7.7)	3 (23.1)	0.120	
	Normal		9 (69.2)	2 (15.4)		
	Excessive		3 (23.0)	8 (61.5)		

MCI: Menstrual cycles interval, MCL: Menstrual cycle length, RMC: Regularity of menstrual cycles.

**Table 4:** Anthropometric characteristics of PCOS patients that received *Ferula assa-foetida *L (Asafoetida) and those who received low dose oral contraceptive (LD) treatments. Data are presented as Mean±SD.

**Variables**	**Group**	**Pre-treatment**	**Post-treatment**	**p value**	
				**With in**	**Between**
BMI (kg/m2)	asafoetida	29.23±5.04	28.80±4.76	0.330	0.854
	LD	26.60±4.05	26.39±4.10	0.333	
WC (cm)	asafoetida	96.93±9.06	95.70±9.59	0.19	0.27
	LD	90.65±8.58	89.64±9.30	0.21	
HC (cm)	asafoetida	110.93±10.01	111.33±9.37	0.806	0.188
	LD	105.15±7.81	104.15±8.32	0.674	

BMI: Body mass index, WC: Waist circumference, HC: Hip circumference

**Table 5:** Hematological and biochemical status of PCOS patients that received *Ferula assa-foetida *L (Asafoetida) and those who received low-dose oral contraceptive (LD) treatments.

**Variables**	**Group**	**Pre-treatment **	**Post-treatment **	**p value**	
				**With in**	**Between**
HB (gr/dl)	asafoetida	13.1±1.09	13.0±0.90	0.509	0.519
	LD	13.6±0.90	13.5±1.15	0.582	
MCV (fL)	asafoetida	83.9±6.91	84.7±4.91	0.875	0.771
	LD	83.2±4.11	84.9±5.97	0.311	
FBS (mg/dl)	asafoetida	93.1±12.50	95.5±10.65	0.220	0.717
	LD	87.2±6.39	90.8±12.99	0.463	
TG (mg/dl)	asafoetida	136.2±68.61	150.6±78.97	0.691	0.911
	LD	107.6±42.82	130.2±54.49	0.023	
CHOL (mg/dl)	asafoetida	164.4±31.00	170.3±26.60	0.256	0.157
	LD	150.2±27.08	174.9±37.04	0.004	
Total testosterone (ng/ml)	asafoetida	0.5±0.28	0.5±0.43	0.480	0.628
	LD	1.0±0.64	0.6±0.34	0.05	
Free testosterone (ng/ml)	asafoetida	1.8±1.44	2.2±1.44	0.256	0.110
	LD	2.1±0.83	1.5±0.89	0.101	
LDL (mg/dl)	asafoetida	96.4±28.63	97.3±25.10	0.414	0.099
	LD	90.3±25.00	106.1±25.75	0.01	
HDL (mg/dl)	asafoetida	47.9±25.80	44.1±13.06	0.443	0.468
	LD	37.0±6.20	41.7±6.91	0.022	
AST(IU/L)	asafoetida	17.0±5.50	18.7±6.82	0.329	0.752
	LD	21.1±10.00	20.6±6.68	0.624	
ALT (IU/L)	asafoetida	20.4±13.03	19.7±12.29	0.865	0.592
	LD	21.8±15.46	22.1±8.58	0.421	
ALP (IU/L)	asafoetida	183.4±45.69	192.7±57.92	0.589	0.229
	LD	184.5±45.65	172.1±60.55	0.675	

Data are presented as Mean±SD. HB: Hemoglobin, MCV: Mean corpuscular volume, FBS: Fasting blood sugar, TG: Triglyceride, CHOL: Cholesterol, LDL: Low-density lipoprotein, HDL: High-density lipoprotein, AST: Aspartate Aminotransferase, ALT: Alanine Aminotransferase, ALP: Alkaline Phosphatase

**Table 6:** The ovarian ultrasound examination, hirsutism status and the incidence of pregnancy (No, %) in PCOS patients that received *Ferula assa-foetida *L (asafoetida) and those who received low dose oral contraceptive (LD) treatments.

**Variables**	**Status**	**Group**	**Pre-treatment**	**Post-treatment**	**p value**	
					**With in**	**Between**
PCO in sonography	Yes	asafoetida	15	11 (73.3)	0.099	0.176
	Yes	LD	13	12 (92.3)	>0.999	
Hirsutism	No	asafoetida	3 (20.0)	5 (33.3)	0.681	>0.999
	Yes		12 (80)	10 (66.6)		
	No	LD	2 (15.4)	4 (30.8)	0.645	
	Yes		11 (84.6)	9 (69.2)		
Area of ovaries (mm^2^)		asafoetida	1879.7±437.91	1762.3±351.23	0.532	0.976
		LD	1775.1±342.72	1750.6±398.68	0.705	
Pregnancy in married patients		asafoetida		7/14 (50.0)		0.019
		LD		0/10 (0)		

## Discussion

The objective of the present study was to compare the therapeutic effect of Asafoetida and low dose contraceptive tablets on PCOS patients. The main demand of married PCOS patients is to become conceived. Our results indicated that half of married patients (7/14) became pregnant within 8 months after receiving Asafoetida; whereas, none of the patients that received LD (0/10 married patients) became pregnant at the same time frame. Seventy percent of women with anovulation have PCOS (Carson and Kallen, 2021) and the incidence of infertility in PCOS women is ten times higher than that of healthy women (Tiwari et al., 2021). Meanwhile, long-term use of synthetic estrogen and progesterone may lead to severe side effects such as infertility (Shukla et al., 2017) and/or decreased conception rate (Silver et al., 2020). 

About 50–60% of PCOS women are obese or overweight (Brennan et al., 2019). Obesity leads to infertility via several mechanisms (Dağ and Dilbaz,, 2015). It contributes to menstrual irregularities and anovulation and reduces conception rate (Zain and Norman, 2008). Weight loss programs have been proven to restore ovulation and menstrual cycles (Silvestris et al., 2018) and improve the chance of conception (Zain and Norman, 2008; Silvestris et al., 2018). In the present study, Asafoetida did not have a significant effect on patients' BMI; however, it was considered an anti-obesity medication (Amalraj and Gopi, 2017; Azizian et al., 2012; Rafiee et al., 2020). 

Another approach to enhance fertility in PCOS patients is to induce ovulation using gonadotropins (Barbieri, 2019). However, such medication may lead to some adverse effects such as ovarian hyperstimulation syndrome (Carson and Kallen, 2021). 

 In the present study, the daily consumption of 1g Asafoetida for 6 weeks was significantly effective for normalizing menstrual cycle intervals for a long period after treatment compared to LD treatment. In one study, the daily consumption of 200 mg oleo-gum resin of Asafoetida for 3 months was not effective to treat oligomenorrhea of PCOS patients (Ghavi et al., 2020). The possible explanation for the difference between the two studies could be related to the dose and/or the type of Asafoetida’s formulation. Further studies on more subjects are required to confirm the results of the present study.

According to the findings of the present trial, the lipid profile of PCOS patients including triglycerides, cholesterol, HDL and LDL significantly increased following LD treatment, which is in agreement with previous studies (Naz et al., 2012; Ismail et al., 2021). Extract of Asafoetida reduced cholesterol, triglycerides, LDL (Azizian et al., 2012; Iranshahi and Alizadeh, 2012), testosterone, AST and ALT (Ayoubi et al., 2013) in rats. However, such changes were not found in the present study. This could be due to species specific difference between humans and rats in responding to Asafoetida. Liver enzymes, before and after treatment with Asafoetida were within the normal range in the present study. This could indicate that the dose and duration of Asafoetida used in this study was not toxic for the patients. Extracts and the oleo-gum resin of Asafoetida have shown dose-dependent cytotoxicity (Bagheri et al., 2010). But the therapeutic dose of Asafoetida used in the present study was within the safe range (0.9-3 g) advised previously (Duke et al., 2002). Asafoetida is considered a hepatoprotective (Silver et al., 2017), antioxidant (Kiasalari et al., 2013), anticarcinogenic (Mokhtareeizadeh and Homayouni Tabrizi, 2021) and anti-cytotoxic treatment (Bagheri et al., 2017). In contrast, the relative risks of hepatocellular carcinoma (Srikanth and Manisree, 2013) and blood pro/antioxidant imbalance (Kowalska and Milnerowicz, 2016) increased in women who receive LD tablets. 

The small sample size of this trial and drug administration for 2 menstrual cycles (6 weeks) were some limitations of the present study.

Asafoetida had the positive impact on the occurrence of regular menstrual cycle in PCOS patients. Moreover, PCOS patients who received Asafoetida had a higher chance to become pregnant.

Asafoetida not only could improve oligomenorrhea, but also maintained menstrual cyclicity for longer period compared to the LD treatment, without displaying LD side effects. Moreover, it increased the possibility of pregnancy in PCOS patients.
